# First Ukrainian Growth References for Height, Weight, and Body Mass Index for Children and Adolescents Aged 7 to 18 Years

**DOI:** 10.1155/2018/9203039

**Published:** 2018-11-11

**Authors:** Serhiy Nyankovskyy, Katarzyna Dereń, Justyna Wyszyńska, Olena Nyankovska, Edyta Łuszczki, Marek Sobolewski, Artur Mazur

**Affiliations:** ^1^Pediatrics Department, Danylo Halytsky L'viv National Medical University, Ukraine; ^2^Medical Faculty, University of Rzeszów, Poland; ^3^Department of Pediatrics and Neonatology. Faculty of Postgraduate Education at Danylo Halytsky L'viv National Medical University, Ukraine; ^4^Faculty of Management, Rzeszów University of Technology, Rzeszów, Poland

## Abstract

*Introduction. *To date, growth centiles of children and adolescents have not been created in Ukraine. Therefore, the aim of this study was to construct reference growth charts for height, weight, and body mass index (BMI) of Ukrainian school-aged children and to compare them with World Health Organization references from 2007 for children's BMI.* Material and Methods*. Among the representative sample of 13,712 students (aged 7 to 18 years) who were included in this study, 6,582 (48%) were boys and 7,130 (52%) were girls. Assessments of height, body mass, and BMI of participants were performed. Reference charts were developed using LMS models within the ChartMaker lms program.* Results*. We present first growth references for height, weight, and body mass index for Ukrainian children and adolescents aged 7 to 18 years. The younger Ukrainian pediatric population (7-13 years) was heavier than population reported in the multiethnic WHO references, while the older (13-18 years) had lower body weight comparing to the same references from WHO.* Conclusions.* The constructed reference growth charts are a benchmark for following secular trends in Ukraine and are also an optimal clinical tool for health care. We recommend national implementation of the Ukrainian reference growth charts for anthropometric measurements.

## 1. Introduction

Childhood is a fundamental phase shaping human development. The health and well-being of children from a given nation reflect both its state of socioeconomic development and the quality of the healthcare system [1]. Somatic growth of children can be used as an indicator of their health, nutritional status, and living standards [2]. Therefore, the governments of many countries regularly collect and analyze anthropometric data regarding the physical development of their population [3–6].

The assessment of height, body mass, and body mass index (BMI) is an important element in the assessment of children's health. The aim of these assessments is to obtain a number of reliable anthropometric measurements and compare them to a reference system (population standards), which reflects the normal variability in relation to age and gender. Growth norms, body weights, and BMI are developed in different countries using various test methods and are periodically updated to reflect changes caused by environmental factors and/or the phenomenon of the secular trend [7, 8]. This phenomenon, observed over the last 100 years, concerns obtaining higher values of final body dimensions between successive generations [9]. The secular trend, which is related to socioeconomic development, is observed in both developed and developing countries [10]. The secular growth trend shows a similar pattern around the world, although its time and velocity may vary [11]. In many developed countries, the secular height trend has reached the plateau phase, while average body mass continues to grow [12]. Due to the phenomenon of the secular trend, population standards need to be updated every 10-15 years [13]. Updated reference growth charts describing the auxological characteristics of the population are particularly useful to the pediatrician.

Due to the lack of national reference systems for the development, growth, and nutritional status of children and youth of school age in Ukraine, pediatricians are forced to use centile charts developed in other countries. Nonetheless, pediatricians are aware that the use of centile charts developed on the basis of studies of other populations can imply nonnegligible biases in their assessment of patient growth due to genetic, environmental, and socioeconomic conditions, lifestyle, and diet.

## 2. Purpose of the Paper

The main aim of this project was to develop Ukrainian growth centile charts of body mass and BMI for the developmental age population. The BMI centile values of 3rd, 85th, and 97th centile (being cut-off points for underweight, overweight and obesity, respectively) were also compared with the same values provided by the World Health Organization (WHO).

## 3. Material and Methods

The study was approved by the regional ethics committee. Written informed consent was obtained from all subjects.

### 3.1. Participants

The study was conducted in randomly selected primary, secondary, and high schools in Ukraine. A multistage random cluster sampling method was used to select the participants aged 7–18 years. Approximately 25,000 children and adolescents were selected from 50 primary, secondary, and high schools from 20 districts of Ukraine. All students from the selected schools were invited to participate in the study, and 15,456 parental approvals were received for participation of their children in the study. Inclusion criteria were as follows: obtaining informed consent from each participant and their parents or guardians, being enrolled in the selected schools, a functional state that allow for self-maintenance of a standing position, not taking medication affecting body weight, and an age between 7 and 18 years.

Out of 15,456 students whose parents gave approval for examination, 1,744 students were excluded from the study for the following reasons: a functional state that did not allow for self-maintenance of a standing position (n=38), chronic disease or medication affecting growth/weight (e.g., asthma, congenital heart disease, diabetes, renal disease, epilepsy, and cerebral palsy) (n=64), age less than 7 years or greater than 18 years (n=125), a lack of desire to participate in the study or a strong pretest anxiety (n=52), and absence from school on assessment days (n=1,465). Ultimately, the study group consisted of 13,712 children and adolescents aged 7.0-18.9 years. Among the representative sample of 13,712 students who were included in this study, 6,582 (48%) were boys and 7,130 (52%) were girls. In the studied population, 46.1% came from rural areas (6,321 of all children and adolescents).

The medical history of participants in the study, including previous and current diseases, as well as medications used, was obtained from the parents of the examined. The general health status of each participant was evaluated by a physician. All measurements were carried out by a team of trained researchers using the same equipment.

The child's exact age was calculated from the difference between the date of the examination and the date of birth. Exact ages were classified into age groups x (where x = 7 to 18) by placement of exact age within the interval (x - 0.5 years, x + 0.5 years). For example, exact ages of 7.6 and 8.4 years would both be classified as belonging to the 8-year age group.

### 3.2. Anthropometrics Measurements

The examinations were carried out in the offices of school nurses in the morning. The tests were carried out by the same team of experienced researchers using the same equipment. For each participant, height and weight were measured. These measurements were made in compliance with WHO recommendations, with the students in their underwear and without shoes [14]. All measurements were taken three times and the mean measurement was recorded in cases of differences.

### 3.3. Body Mass, Body Height, and BMI

The body weight of study participants was evaluated using the electronic scales RADWAG WPT 60/150 (RADWAG) with an accuracy of +/- 50 g. The test was performed without footwear, in underwear, after emptying the bladder. Body height was assessed using a measuring instrument attached to the scales, in a standing upright position without footwear. BMI was calculated as body mass in kilograms divided by the square of the height in meters (kg/m^2^).

### 3.4. Data Analysis

Statistical analysis was carried out using STATISTICA 10.0 and EXCEL 2010 software. Centile charts were developed using specialized LMS models using lms ChartMaker software. Using curves modeled with LMS, the mean annual increases in body height were also determined between 7 and 18 years of age. The level of the 3rd, 85th, and 97th percentile (using cut-off point recommendations of the International Obesity Task Force [15]) and the results obtained using LMS curves were compared with analogous values given by the WHO [16] graphically for both boys and girls.

## 4. Results

Growth references presented are based on a representative sample of 13,712 school-age children and adolescents, including 6,582 boys and 7,130 girls. The characteristics of height, weight, and BMI with regard to sex are presented in Table 1.

**Figure 1:**
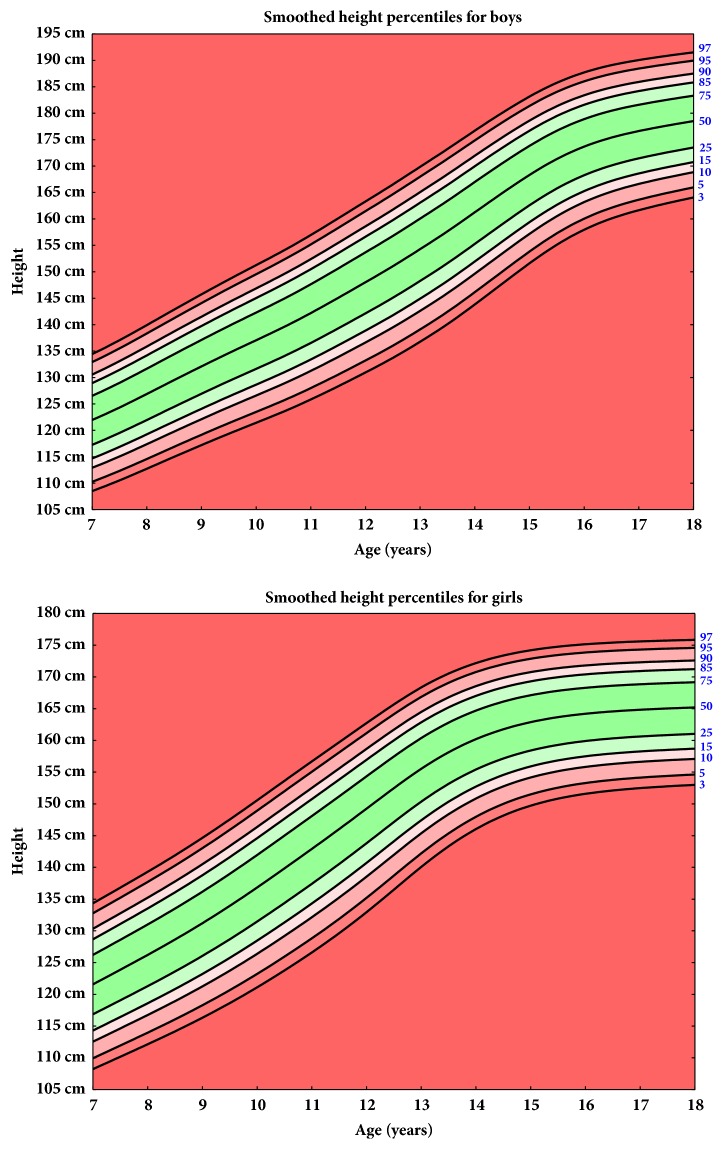
Smoothed height percentiles for boys and girls.

**Figure 2:**
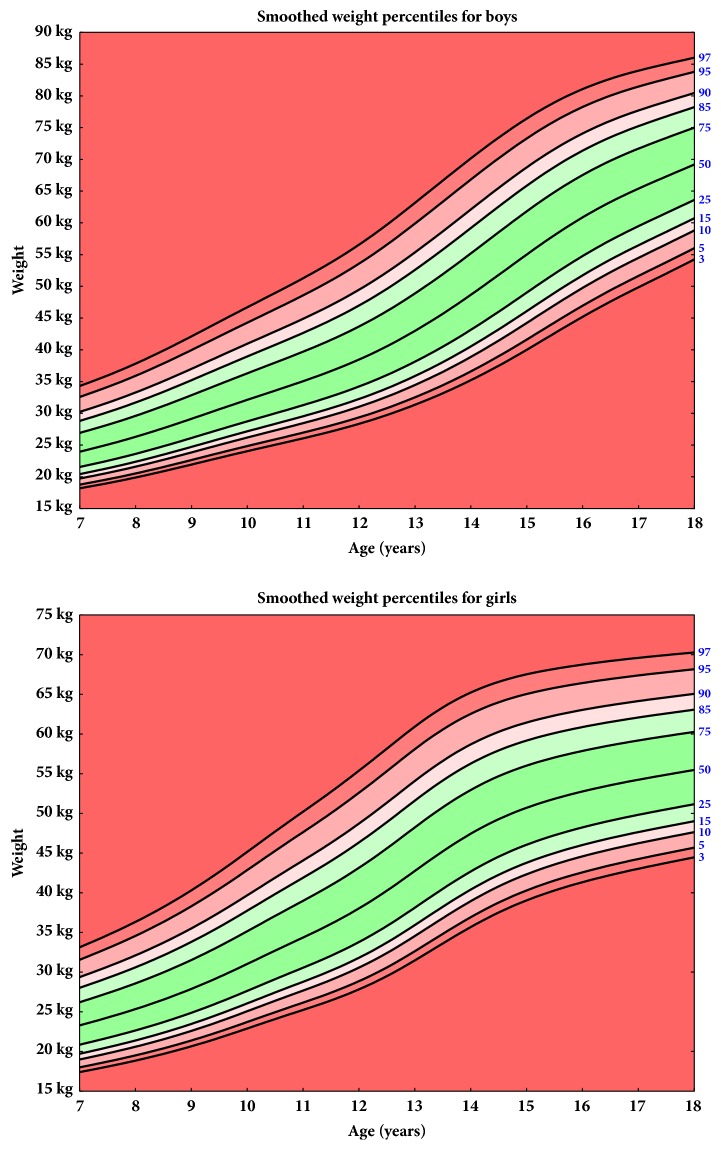
Smoothed weight percentiles for boys and girls.

**Figure 3:**
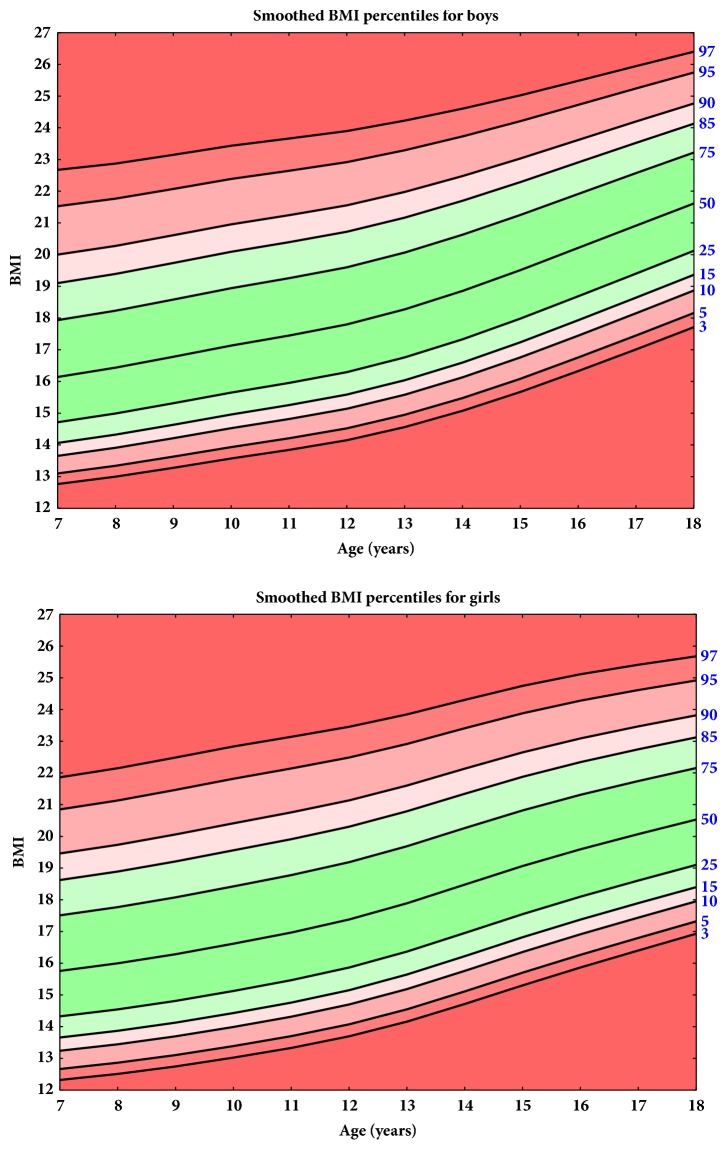
Smoothed BMI percentiles for boys and girls.

The current height, body weight, and BMI percentiles for the school-age children are shown in Figures 1, 2, and 3, respectively.

**Figure 4:**
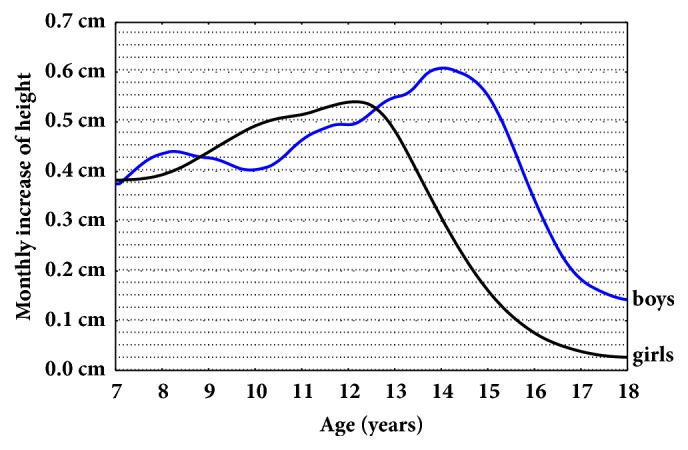
Monthly increases in height for children and adolescents aged 7-18.

Using the height values of children between 7 and 18 years of age modeled with LMS curves, average annual increases in body height were determined. The rate of body height increase during a year for girls and boys from the Ukrainian population between 7 and 18 years is shown in Figure 4. In the population sample of healthy Ukrainian children, the acceleration in the rate of body height increase is the highest in girls aged 12 and approximately two years later for boys. The greatest acceleration in the rate of body height is achieved in boys aged 14 years 2 months (approximately 0.61 cm monthly) and in girls aged 12 years 3 months (approximately 0.54 cm monthly). When analyzing the annual increases in body height, it was found that the periods of the highest annual growth for boys are from 12 years 7 months to 13 years 8 months and the annual increase in this period is on average 7.22 cm. For girls it is between 11 years 7 months and 12 years 6 months, and the annual increase is 6.44 cm on average.

**Figure 5:**
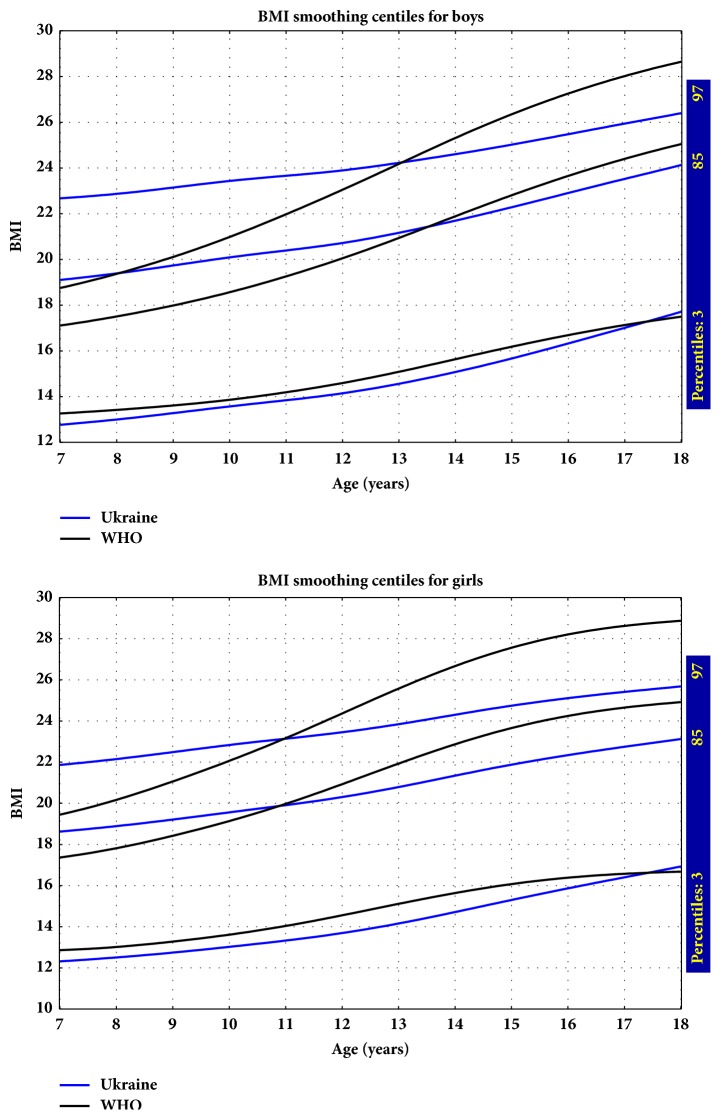
Comparison of 3rd, 85th, and 97th BMI centiles for boys and girls: Ukraine versus WHO.

*Comparison of WHO References and Ukrainian References for Children Aged 7-18.* Figure 5 shows comparison of the 3rd, 85th, and 97th percentiles of BMI for Ukraine and data provided by the WHO. At the 3rd percentile, the curves are similar; however, a slightly lower value in the Ukrainian population indicates that the prevalence of underweight is higher than in the WHO reference population. For the 85th and 97th percentiles, the values were higher for the Ukrainian population between 7 years and around 13-13.5 years for boys and 11 for girls. The opposite situation occurs in the case of older participants from about an age of 13 for boys and 11 years for girls: the 85th and 97th BMI percentiles according to WHO references are higher than the values calculated for the Ukrainian community.

## 5. Discussion

Assessment of the physical development of children and adolescents requires the analysis of a set of morphological features including body measurements and then comparing the results with current population standards serving as a reference system. Developmental parameters such as height, body weight, and BMI are generally changing with the age of the child. Due to the continuous process of a child's development, height, body mass, and BMI are specific to age and sex; therefore, the current centile charts should be used in the developmental age population [17].

The cross-sectional references for height, weight, and BMI presented in this paper are the first references to apply to the whole Ukrainian population from 7 to 18 years of age. There are no growth references in Ukraine that would allow for referencing the results of a child's development assessment. This led to a paradoxical situation in which the results of the child's assessment were compared to the standards developed for different populations in other countries. The implementation of the standards developed in other countries could have considerable clinical implications and result in unnecessary referrals to pediatric departments as a larger number of Ukrainian children would be considered abnormal.

Currently, in a population of healthy Ukrainian children, the acceleration of the growth rate begins in girls aged 8 years 3 months and for boys aged 10 years 3 months. Final height was 178.4 cm for boys and 164.2 cm for girls, indicating that boys and girls are taller by about 1.9 cm and 1 cm, respectively, than the WHO reference. The resulting sex difference of 14.2 cm was similar to that found in the WHO reference (13.3 cm) [16].

Comparison of the 3rd percentiles of BMI for Ukraine and data provided by the WHO indicated that the prevalence of underweight in the Ukrainian pediatric population is higher than in the WHO reference population. Comparing the BMI percentiles for boys and girls, we found that the problem of underweight is greater among girls than boys. Being underweight among children and adolescents is associated with an increased susceptibility to fat accumulation, lower fat oxidation, lower energy expenditure, a higher risk of insulin resistance in adulthood, dyslipidemia, and hypertension [18]. Globally, in 2016, the prevalence of moderate and severe underweight was 8.4% in girls and 12.4% in boys [19]. According to Rokholm et al., the rise in excess body weight in children and adolescents has plateaued in high-income countries but continues in low-income and middle-income countries, and the relatively rapid transition from underweight to excessive body weight in low-income and middle-income countries has been noted [20].

Comparison of the 85th and 97th percentiles of BMI for Ukraine with data provided by the WHO showed that among the younger Ukrainian population (between 7 and 13-13.5 years for boys and 11 years for girls) both the 85th and 97th centiles of BMI in Ukraine are clearly higher than the corresponding WHO centiles; evidently, according to the WHO references in Ukraine, younger children are at a higher risk of overweight and obesity. The opposite situation occurs in the case of older participants from about age of 13 years for boys and 11 years for girls. The 85th and 97th BMI percentiles according to WHO references are higher than the values calculated for the Ukrainian community. This suggests that the problem of obesity and overweight affects Ukrainian adolescents to a lesser extent than the WHO reference population.

According to Maydannyk et al., the prevalence of obesity among Ukrainian adolescents has increased 2.5 times over the past ten years [21]. We speculate that the increase in BMI in the younger Ukrainian population is caused by an increase in the socioeconomic development of Ukraine. Due to higher standards of living, higher social class, more frequent fast food consumption, and declining physical activity level, Ukrainian pediatric population are reaching a higher prevalence of obesity earlier in the life course. Older Ukrainian population has lower prevalence of obesity. This can be explained by fact that obesity is established very early in life and that it basically tracks through adolescence to adulthood. Children who have excessive body weight stay obese or overweight in adolescence, and those whose weight is healthy do not become obese [22].

Obesity is a serious problem in both developed and developing countries [23]. Obesity, as a complex disorder, is strongly related to lifestyle and associated with age, sex, family income, and urbanization. For children, the risk of obesity is associated with the educational and BMI levels of their parents, high birth weight, living in a urban area, and eating food that was not prepared at home [24]. Although high prevalence of obesity is a problem of all socioeconomic groups, the association of obesity with socioeconomic factors is different in developed and developing countries [25]. The relationship between socioeconomic status and obesity in the pediatric population has been well documented; however, inconsistent results were noticed considering the country's income [26]. Most studies indicate that obesity in children and adults from low and middle-income countries has a strong positive association with socioeconomic status [27]. In turn, an inverse association is observed in high-income countries [28].

A higher prevalence of excessive body weight in the younger population from Ukraine may result from a combination of low levels of physical activity and unhealthy nutrition. Increases in socioeconomic development are associated with changes in lifestyle of the population and better access to high-calorie foods. Moreover, as a result of technological progress, a significant reduction in energy expenditure associated with daily activities contributes to higher frequency of sedentary habits [29].

Excessive body weight is a growing public health problem in many countries and has significant consequences due to higher morbidity [30], negative impact on quality of life [31], and higher healthcare expenditures [32]. Health in childhood and adolescence is the basis of health in adulthood. Epidemiological research has demonstrated that obesity and low levels of physical activity contribute significantly to the prevalence of cardiovascular disease. In addition, it has been found that the conditions of overweight and obesity tend to remain stable from birth through childhood and adolescence to adulthood [33]. Therefore, early diagnosis of childhood obesity is essential to implementation of treatment. Moreover recognizing the factors contributing to childhood obesity in relation to biological, socioeconomic, and associated lifestyle factors may provide a framework for policymakers to develop a health strategy for preventing obesity-related health consequences.

## 6. Conclusions

Reference growth curves for height, weight, and BMI that were constructed from measurements of 13,712 school-age children and adolescents allow for early detection of disorders in physical development and nutrition. The presented curves might be an optimal clinical tool for healthcare in Ukraine.

## Figures and Tables

**Table 1:** The characteristics of height, weight, and BMI with regard to sex.

**Age ** **[y]**	**Height [cm]**								**Weight [kg]**								**BMI [kg/m** ^**2**^ **]**							
	**Boys**				**Girls**				**Boys**				**Girls**				**Boys**				**Girls**			
	**Min**	**Max**	**Mean**	**SD**	**Min**	**Max**	**Mean**	**SD**	**Min**	**Max**	**Mean**	**SD**	**Min**	**Max**	**Mean**	**SD**	**Min**	**Max**	**Mean**	**SD**	**Min**	**Max**	**Mean**	**SD**
**7**	100.0	145.0	122.0	7.01	95.0	150.0	121.9	7.13	16.0	40.0	24.9	4.32	15.0	45.0	24.0	4.22	11.7	29.0	16.7	2.73	10.2	27.5	16.1	2.47

**8**	104.0	156.0	126.9	7.22	104.0	150.0	126.4	6.83	16.0	51.0	26.9	4.87	15.0	48.0	26.1	4.70	10.7	30.0	16.7	2.69	10.9	28.5	16.3	2.41

**9**	106.0	155.0	132.1	7.60	105.0	155.0	130.9	7.52	18.0	55.0	30.1	5.59	16.0	50.0	28.7	5.58	11.5	32.0	17.2	2.66	10.2	30.0	16.7	2.77

**10**	110.0	160.0	137.0	7.78	110.0	160.0	136.5	8.25	20.0	60.0	33.1	6.14	20.0	56.0	31.8	5.77	10.4	28.8	17.6	2.57	11.2	29.2	17.0	2.67

**11**	115.0	170.0	142.0	8.21	116.0	167.0	142.9	8.19	21.0	67.0	36.2	6.80	21.0	66.0	35.4	6.93	11.2	27.6	17.9	2.67	11.6	34.0	17.3	2.72

**12**	120.0	178.0	147.8	8.99	120.0	177.0	148.2	8.37	23.0	79.0	39.5	7.99	20.0	78.0	39.0	7.76	12.4	36.7	18.0	2.76	11.7	32.9	17.7	2.69

**13**	125.0	183.0	153.9	9.51	120.0	176.0	155.2	8.57	25.0	87.0	44.4	9.17	26.0	70.0	43.8	8.10	13.0	36.1	18.6	2.77	12.9	29.6	18.1	2.61

**14**	130.0	187.0	160.9	9.17	135.0	180.0	160.0	6.85	30.0	92.0	49.9	9.55	28.0	78.0	48.4	7.96	12.9	32.2	19.2	2.58	12.4	30.7	18.9	2.64

**15**	140.0	198.0	167.7	9.12	141.0	180.0	163.0	6.27	30.0	104.0	55.9	10.03	30.0	83.0	51.6	7.59	13.8	32.0	19.8	2.52	11.7	29.4	19.4	2.48

**16**	146.0	197.0	173.6	8.04	142.0	182.0	163.7	6.10	30.0	100.0	61.3	9.45	36.0	90.0	53.1	7.01	11.2	28.1	20.3	2.29	14.9	33.1	19.8	2.34

**17**	157.0	195.0	176.8	6.54	145.0	183.0	164.6	5.98	37.0	104.0	66.6	8.95	35.0	85.0	54.9	7.27	13.4	32.1	21.3	2.42	13.3	30.5	20.3	2.49

**18**	160.0	194.0	178.4	6.81	147.0	181.0	164.2	5.77	48.0	95.0	68.7	8.05	40.0	80.0	55.7	7.07	15.2	26.6	21.6	2.05	15.6	29.0	20.6	2.39

## Data Availability

The data used to support the findings of this study are included within the article.

## Supplementary Materials

Supplementary Materials Numerical values of height, weight, and BMI centiles by sex and age are presented in supplementary Tables S1, S2, and S3, respectively.
