# Supplementary material for: First Ukrainian Growth References for Height, Weight, and Body Mass Index for Children and Adolescents Aged 7 to 18 Years
Source: Biomed Res Int. 2018 Nov 11;2018:9203039. doi: 10.1155/2018/9203039 (PMC6252215; doi:10.1155/2018/9203039)
Supplement: Supplementary Materials — Numerical values of height, weight, and BMI centiles by sex and age are presented in supplementary Tables S1, S2, and S3, respectively. [file 9203039.f1.docx]

**TABLE S1** Smoothed height percentiles for boys and girls

| **Age [years]** | **Smoothed height percentiles – boys** | | | | | | | | | | |
| --- | --- | --- | --- | --- | --- | --- | --- | --- | --- | --- | --- |
|  | **3** | **5** | **10** | **15** | **25** | **50** | **75** | **85** | **90** | **95** | **97** |
| **7** | 108.5 | 110.3 | 112.9 | 114.7 | 117.3 | 122.0 | 126.5 | 128.9 | 130.5 | 132.9 | 134.4 |
| **8** | 112.7 | 114.6 | 117.4 | 119.2 | 122.0 | 126.9 | 131.7 | 134.2 | 135.9 | 138.3 | 139.9 |
| **9** | 117.2 | 119.2 | 122.1 | 124.1 | 126.9 | 132.1 | 137.1 | 139.7 | 141.5 | 144.1 | 145.7 |
| **10** | 121.4 | 123.5 | 126.6 | 128.6 | 131.6 | 137.0 | 142.3 | 145.0 | 146.9 | 149.5 | 151.3 |
| **11** | 125.8 | 128.0 | 131.3 | 133.4 | 136.5 | 142.2 | 147.6 | 150.5 | 152.4 | 155.2 | 157.0 |
| **12** | 131.0 | 133.2 | 136.6 | 138.9 | 142.1 | 148.0 | 153.7 | 156.6 | 158.6 | 161.5 | 163.3 |
| **13** | 136.8 | 139.1 | 142.6 | 144.9 | 148.3 | 154.3 | 160.1 | 163.1 | 165.1 | 168.0 | 169.9 |
| **14** | 143.8 | 146.1 | 149.6 | 151.9 | 155.3 | 161.3 | 167.0 | 170.0 | 172.0 | 174.9 | 176.7 |
| **15** | 151.6 | 153.8 | 157.2 | 159.4 | 162.6 | 168.3 | 173.8 | 176.7 | 178.6 | 181.4 | 183.2 |
| **16** | 158.0 | 160.0 | 163.2 | 165.3 | 168.3 | 173.7 | 178.9 | 181.6 | 183.4 | 186.0 | 187.7 |
| **17** | 161.7 | 163.6 | 166.6 | 168.6 | 171.5 | 176.6 | 181.6 | 184.2 | 185.9 | 188.4 | 190.1 |
| **18** | 164.1 | 166.0 | 168.8 | 170.8 | 173.5 | 178.5 | 183.3 | 185.8 | 187.5 | 189.9 | 191.5 |
| **Age [years]** | **Smoothed height percentiles – girls** | | | | | | | | | | |
|  | **3** | **5** | **10** | **15** | **25** | **50** | **75** | **85** | **90** | **95** | **97** |
| **7** | 108.2 | 109.9 | 112.6 | 114.3 | 116.9 | 121.6 | 126.2 | 128.7 | 130.3 | 132.8 | 134.3 |
| **8** | 112.1 | 114.0 | 116.7 | 118.6 | 121.3 | 126.2 | 131.0 | 133.5 | 135.2 | 137.7 | 139.3 |
| **9** | 116.3 | 118.3 | 121.2 | 123.2 | 126.1 | 131.2 | 136.2 | 138.8 | 140.5 | 143.0 | 144.6 |
| **10** | 121.1 | 123.2 | 126.4 | 128.5 | 131.5 | 136.8 | 141.9 | 144.6 | 146.4 | 148.9 | 150.6 |
| **11** | 126.6 | 128.8 | 132.1 | 134.3 | 137.4 | 142.9 | 148.1 | 150.7 | 152.5 | 155.1 | 156.7 |
| **12** | 132.9 | 135.2 | 138.5 | 140.7 | 143.8 | 149.2 | 154.3 | 156.9 | 158.7 | 161.1 | 162.7 |
| **13** | 140.1 | 142.2 | 145.4 | 147.4 | 150.4 | 155.5 | 160.4 | 162.8 | 164.5 | 166.8 | 168.3 |
| **14** | 146.0 | 148.0 | 150.9 | 152.7 | 155.4 | 160.2 | 164.7 | 167.0 | 168.6 | 170.8 | 172.2 |
| **15** | 149.7 | 151.5 | 154.2 | 155.9 | 158.4 | 162.9 | 167.1 | 169.3 | 170.8 | 172.9 | 174.2 |
| **16** | 151.6 | 153.3 | 155.8 | 157.5 | 159.9 | 164.2 | 168.3 | 170.4 | 171.8 | 173.8 | 175.1 |
| **17** | 152.5 | 154.1 | 156.6 | 158.3 | 160.6 | 164.8 | 168.8 | 170.9 | 172.3 | 174.3 | 175.6 |
| **18** | 153.0 | 154.6 | 157.1 | 158.7 | 161.0 | 165.2 | 169.1 | 171.2 | 172.6 | 174.6 | 175.8 |

**TABLE S2** Smoothed weight percentiles for boys and girls

| **Age [years]** | **Smoothed weight percentiles – boys** | | | | | | | | | | |
| --- | --- | --- | --- | --- | --- | --- | --- | --- | --- | --- | --- |
|  | **3** | **5** | **10** | **15** | **25** | **50** | **75** | **85** | **90** | **95** | **97** |
| **7** | 18.2 | 18.8 | 19.7 | 20.4 | 21.5 | 24.0 | 26.9 | 28.8 | 30.2 | 32.6 | 34.3 |
| **8** | 19.9 | 20.5 | 21.6 | 22.4 | 23.6 | 26.3 | 29.6 | 31.7 | 33.3 | 35.9 | 37.8 |
| **9** | 21.9 | 22.7 | 23.8 | 24.7 | 26.1 | 29.1 | 32.9 | 35.2 | 37.0 | 40.0 | 42.1 |
| **10** | 24.1 | 24.9 | 26.2 | 27.2 | 28.7 | 32.1 | 36.3 | 39.0 | 41.0 | 44.3 | 46.7 |
| **11** | 26.1 | 27.0 | 28.5 | 29.5 | 31.3 | 35.1 | 39.7 | 42.7 | 44.9 | 48.6 | 51.3 |
| **12** | 28.4 | 29.4 | 31.0 | 32.3 | 34.2 | 38.5 | 43.7 | 47.0 | 49.5 | 53.6 | 56.6 |
| **13** | 31.4 | 32.5 | 34.5 | 35.9 | 38.1 | 43.0 | 48.9 | 52.6 | 55.4 | 59.9 | 63.2 |
| **14** | 35.2 | 36.6 | 38.9 | 40.5 | 43.2 | 48.7 | 55.2 | 59.2 | 62.1 | 66.8 | 70.2 |
| **15** | 40.0 | 41.6 | 44.2 | 46.1 | 49.0 | 55.0 | 61.8 | 65.9 | 68.8 | 73.3 | 76.4 |
| **16** | 45.2 | 47.0 | 49.8 | 51.8 | 54.8 | 60.9 | 67.6 | 71.4 | 74.1 | 78.3 | 81.1 |
| **17** | 49.9 | 51.7 | 54.5 | 56.5 | 59.5 | 65.4 | 71.7 | 75.2 | 77.7 | 81.5 | 84.0 |
| **18** | 54.2 | 56.0 | 58.8 | 60.7 | 63.6 | 69.2 | 75.0 | 78.2 | 80.4 | 83.8 | 86.0 |
| **Age [years]** | **Smoothed weight percentiles – girls** | | | | | | | | | | |
|  | **3** | **5** | **10** | **15** | **25** | **50** | **75** | **85** | **90** | **95** | **97** |
| **7** | 17.4 | 18.0 | 19.0 | 19.7 | 20.9 | 23.3 | 26.2 | 28.0 | 29.4 | 31.6 | 33.1 |
| **8** | 18.9 | 19.5 | 20.6 | 21.4 | 22.6 | 25.3 | 28.6 | 30.6 | 32.1 | 34.6 | 36.3 |
| **9** | 20.7 | 21.4 | 22.6 | 23.5 | 24.9 | 27.9 | 31.5 | 33.8 | 35.5 | 38.3 | 40.3 |
| **10** | 22.9 | 23.7 | 25.1 | 26.1 | 27.6 | 31.0 | 35.2 | 37.8 | 39.7 | 42.9 | 45.2 |
| **11** | 25.2 | 26.2 | 27.7 | 28.8 | 30.6 | 34.4 | 39.0 | 41.9 | 44.1 | 47.6 | 50.2 |
| **12** | 27.8 | 28.9 | 30.6 | 31.8 | 33.8 | 38.0 | 43.1 | 46.3 | 48.7 | 52.6 | 55.4 |
| **13** | 31.5 | 32.6 | 34.5 | 35.9 | 38.1 | 42.7 | 48.2 | 51.6 | 54.1 | 58.1 | 60.9 |
| **14** | 35.7 | 36.9 | 39.0 | 40.4 | 42.7 | 47.5 | 53.0 | 56.3 | 58.7 | 62.6 | 65.2 |
| **15** | 39.1 | 40.3 | 42.3 | 43.8 | 46.1 | 50.7 | 56.0 | 59.2 | 61.5 | 65.0 | 67.5 |
| **16** | 41.3 | 42.6 | 44.6 | 46.0 | 48.2 | 52.8 | 57.9 | 60.9 | 63.0 | 66.4 | 68.7 |
| **17** | 43.0 | 44.3 | 46.2 | 47.6 | 49.8 | 54.2 | 59.2 | 62.1 | 64.2 | 67.4 | 69.6 |
| **18** | 44.4 | 45.7 | 47.6 | 49.0 | 51.1 | 55.5 | 60.3 | 63.1 | 65.0 | 68.2 | 70.3 |

**TABLE S3** Smoothed BMI percentiles for boys and girls

| **Age [years]** | **Smoothed BMI percentiles – boys** | | | | | | | | | | |
| --- | --- | --- | --- | --- | --- | --- | --- | --- | --- | --- | --- |
|  | **3** | **5** | **10** | **15** | **25** | **50** | **75** | **85** | **90** | **95** | **97** |
| **7** | 12.8 | 13.1 | 13.7 | 14.1 | 14.7 | 16.1 | 17.9 | 19.1 | 20.0 | 21.5 | 22.7 |
| **8** | 13.0 | 13.3 | 13.9 | 14.3 | 15.0 | 16.4 | 18.2 | 19.4 | 20.3 | 21.8 | 22.9 |
| **9** | 13.3 | 13.6 | 14.2 | 14.6 | 15.3 | 16.8 | 18.6 | 19.7 | 20.6 | 22.1 | 23.1 |
| **10** | 13.6 | 13.9 | 14.5 | 15.0 | 15.7 | 17.1 | 18.9 | 20.1 | 21.0 | 22.4 | 23.4 |
| **11** | 13.8 | 14.2 | 14.8 | 15.3 | 16.0 | 17.4 | 19.3 | 20.4 | 21.2 | 22.6 | 23.7 |
| **12** | 14.1 | 14.5 | 15.1 | 15.6 | 16.3 | 17.8 | 19.6 | 20.7 | 21.6 | 22.9 | 23.9 |
| **13** | 14.6 | 15.0 | 15.6 | 16.0 | 16.8 | 18.3 | 20.1 | 21.2 | 22.0 | 23.3 | 24.2 |
| **14** | 15.1 | 15.5 | 16.1 | 16.6 | 17.3 | 18.9 | 20.6 | 21.7 | 22.5 | 23.7 | 24.6 |
| **15** | 15.7 | 16.1 | 16.8 | 17.2 | 18.0 | 19.5 | 21.3 | 22.3 | 23.0 | 24.2 | 25.0 |
| **16** | 16.3 | 16.8 | 17.4 | 17.9 | 18.7 | 20.2 | 21.9 | 22.9 | 23.6 | 24.7 | 25.5 |
| **17** | 17.0 | 17.5 | 18.2 | 18.6 | 19.4 | 20.9 | 22.6 | 23.5 | 24.2 | 25.2 | 25.9 |
| **18** | 17.7 | 18.2 | 18.9 | 19.4 | 20.1 | 21.6 | 23.2 | 24.1 | 24.8 | 25.7 | 26.4 |
| **Age [years]** | **Smoothed BMI percentiles – girls** | | | | | | | | | | |
|  | **3** | **5** | **10** | **15** | **25** | **50** | **75** | **85** | **90** | **95** | **97** |
| **7** | 12.3 | 12.7 | 13.2 | 13.7 | 14.3 | 15.8 | 17.5 | 18.6 | 19.5 | 20.8 | 21.9 |
| **8** | 12.5 | 12.9 | 13.4 | 13.9 | 14.5 | 16.0 | 17.8 | 18.9 | 19.7 | 21.1 | 22.1 |
| **9** | 12.7 | 13.1 | 13.7 | 14.1 | 14.8 | 16.3 | 18.1 | 19.2 | 20.1 | 21.5 | 22.5 |
| **10** | 13.0 | 13.4 | 14.0 | 14.4 | 15.1 | 16.6 | 18.4 | 19.6 | 20.4 | 21.8 | 22.8 |
| **11** | 13.3 | 13.7 | 14.3 | 14.8 | 15.5 | 17.0 | 18.8 | 19.9 | 20.8 | 22.1 | 23.1 |
| **12** | 13.7 | 14.1 | 14.7 | 15.1 | 15.9 | 17.4 | 19.2 | 20.3 | 21.1 | 22.5 | 23.5 |
| **13** | 14.2 | 14.5 | 15.2 | 15.6 | 16.4 | 17.9 | 19.7 | 20.8 | 21.6 | 22.9 | 23.8 |
| **14** | 14.7 | 15.1 | 15.8 | 16.2 | 17.0 | 18.5 | 20.3 | 21.3 | 22.1 | 23.4 | 24.3 |
| **15** | 15.3 | 15.7 | 16.4 | 16.8 | 17.5 | 19.1 | 20.8 | 21.9 | 22.6 | 23.9 | 24.7 |
| **16** | 15.9 | 16.3 | 16.9 | 17.4 | 18.1 | 19.6 | 21.3 | 22.3 | 23.1 | 24.3 | 25.1 |
| **17** | 16.4 | 16.8 | 17.4 | 17.9 | 18.6 | 20.1 | 21.7 | 22.7 | 23.5 | 24.6 | 25.4 |
| **18** | 16.9 | 17.3 | 18.0 | 18.4 | 19.1 | 20.5 | 22.2 | 23.1 | 23.8 | 24.9 | 25.7 |
